# Disentangling Elemental Roles in an Amorphous Medium‐Entropy Electrocatalyst for Oxygen Evolution

**DOI:** 10.1002/advs.77381

**Published:** 2026-08-29

**Authors:** Jinhu Wu, Xianjun Cao, Cheng Gong, Dongfang Li, Yaojie Lei, Daojin Zhou, Pengpeng Zhang, Zeliang Wu, Bin Yu, Xingwang Peng, Bernt Johannessen, Somnath Chanda Roy, Jinqiang Zhang, Hao Liu, Yufei Zhao

**Affiliations:** ^1^ Joint International Laboratory on Environmental and Energy Frontier Materials School of Environmental and Chemical Engineering Shanghai University Shanghai China; ^2^ School of Mathematical and Physical Sciences Faculty of Science University of Technology Sydney Sydney New South Wales Australia; ^3^ State Key Laboratory of Chemical Resource Engineering College of Chemistry Beijing University of Chemical Technology Beijing China; ^4^ Australian Synchrotron ANSTO Melbourne Victoria Australia; ^5^ Department of Physics Semiconducting Oxide Materials Nanostructures and Tailored Heterojunctions Indian Institute of Technology Madras Chennai India

**Keywords:** amorphous structure, element roles, medium entropy alloys, oxygen evolution reaction

## Abstract

Understanding elemental cooperation in high/medium‐entropy electrocatalysts is essential for rational design but remains challenging due to their compositional complexity. Herein, an amorphous medium‐entropy catalyst, FeCoNiMo‐a, with a nanoring architecture, is developed as a highly active and durable oxygen evolution catalyst. Operando spectroscopic investigations uncover a clear division of elemental functions within the disordered framework. Cobalt is identified as the dominant active centre, forming high‐valence oxyhydroxide species during operation, while iron and molybdenum synergistically regulate the electronic structure and stabilize these oxidized intermediates. In contrast, nickel remains largely metallic, ensuring efficient charge transport and structural robustness. Through these cooperative effects, FeCoNiMo‐a achieves only 190 mV overpotential at 10 mA cm^−2^ in 1 m KOH. When integrated into an anion exchange membrane (AEM) water electrolyser, FeCoNiMo‐a delivers current densities of 1 and 5 A cm^−2^ at 1.6 and 1.9 V, respectively, and operates stably for over 600 h at 1 A cm^−2^. Further theoretical investigations confirm that Mo incorporation optimizes *OH adsorption and deprotonation, while the amorphous configuration enhances orbital hybridization, facilitating O─O bond cleavage and lattice oxygen participation. This study reveals element‐specific cooperation in medium‐entropy catalysts, guiding the design of non‐precious OER catalysts for practical alkaline water electrolysis.

## Graphical abstract

An amorphous medium‐entropy FeCoNiMo‐a catalyst with a unique nanoring architecture is synthesized via a solvothermal method. The catalyst exhibits exceptional stability in anion exchange membrane water electrolysis, maintaining stable operation for over 600 h at 1 A cm^−2^.

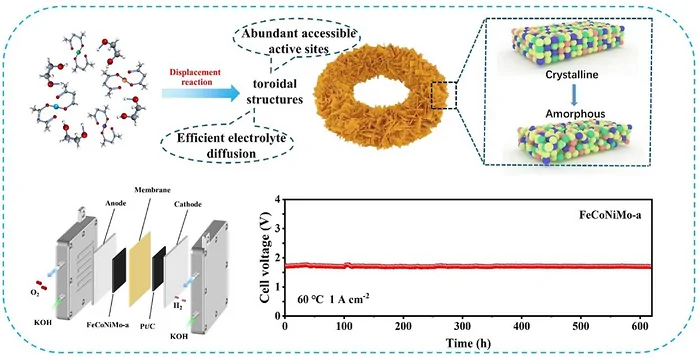

## 1 Introduction

As the global drive toward carbon neutrality accelerates, green hydrogen produced using renewable energy sources is emerging as a pivotal energy vector [1, 2]. Water electrolysis offers an effective approach for converting intermittent renewable power, such as solar and wind, into storable hydrogen [3, 4]. Among the existing technologies, alkaline water electrolysis remains the most widely adopted due to its technological maturity, operational stability, and cost‐effectiveness [5, 6]. Electrocatalytic water splitting comprises the cathodic hydrogen evolution reaction (HER) and the anodic oxygen evolution reaction (OER). However, the overall efficiency of the process is fundamentally limited by the sluggish OER kinetics, which involve a complex four‐electron‐proton coupling process. State‐of‐the‐art OER catalysts are still dominated by noble‐metal‐based materials (e.g., Ir‐ or Ru‐based oxides) [7, 8, 9], and their high cost and limited natural abundance present major barriers to large‐scale deployment. Consequently, the development of highly active, durable, and earth‐abundant OER electrocatalysts has become a central research priority for enabling cost‐effective green hydrogen production and accelerating the transition toward a sustainable energy future [10, 11].

Medium‐entropy materials (MEMs) have recently emerged as a transformative class of catalysts, offering a fundamentally new approach to overcoming the intrinsic limitations of conventional single‐ or binary‐component systems [12, 13]. Composed of three to four elements in near‐equimolar ratios, or quaternary solid solutions with sufficiently high configurational entropy [14, 15, 16], MEMs possess highly disordered atomic arrangements and random elemental distributions that confer exceptional structural diversity, compositional tunability, and stability [17, 18]. This multicomponent environment enables broadened electronic states, tunable d‐band centres, and heterogeneous local coordination environments, optimizing the adsorption energetics of key OER intermediates (*OH, *O, and *OOH) [19, 20]. Intrinsic lattice distortion and the cocktail effect further generate a high density of catalytically active sites and modulate metal‐oxygen covalency, thereby accelerating reaction kinetics. In particular, incorporation of high‐valence metal cations (e.g., W^6+^, V^5+^, and Mo^6+^) plays a critical role in regulating catalytic activity by inducing directional electron transfer from neighboring active metals (such as Fe, Ni, or Co) through M─O─M bonding or electronic polarization, effectively lowering the d‐band centre and balancing intermediate binding strengths [21, 22]. Moreover, the large ionic radii and distinct coordination preferences of high‐valence metals introduce local geometric distortion, further refining reaction pathways. Beyond compositional engineering, amorphous MEM architecture provides abundant coordinatively unsaturated sites and structural defects, while their isotropic and flexible atomic frameworks enable dynamic surface reconstruction under electrochemical processes, forming metastable yet highly active catalytic states. Therefore, these features position MEMs as a versatile and powerful catalyst platform for OER [23].

Despite the remarkable performance advantages of MEMs, one of the most fundamental challenges in this field lies in disentangling the specific roles of individual elements within their multicomponent and strongly coupled frameworks [24, 25]. Unlike conventional catalysts, where active sites can often be clearly identified, the intrinsic compositional complexity of MEMs makes it exceedingly difficult to quantitatively assign catalytic function to each constituent element. In principle, different elements within MEMs may serve distinct roles, including acting as catalytic active sites, regulating electronic conductivity, enhancing corrosion resistance, or stabilizing the crystal structure [26]. For oxygen evolution catalysis, both noble metals (e.g., Ir, Ru) [27, 28] and earth‐abundant transition metals (e.g., Fe, Co, and Ni) [29, 30] have been identified as potential active centres, while other elements such as V or Mo often function as promoters by modulating the local electronic structure [15, 31], coordination environment, and adsorption energetics of neighboring active sites. Additional stabilizing elements (e.g., Cr) are frequently introduced to suppress dissolution and maintain structural integrity under harsh electrochemical conditions [32]. However, although isolated studies have attempted to distinguish active sites from promoters or other functions, it remains rare and experimentally challenging to clearly and unambiguously resolve the individual contributions of multiple elements coexisting in the same MEM, particularly under dynamic operating conditions. This persistent “mechanistic black box” severely limits the rational design and optimization of MEMs and underscores the urgent need for systematic strategies capable of decoupling elemental functions and tracking their evolution during catalysis [33].

Herein, an amorphous quaternary medium‐entropy catalyst, FeCoNiMo‐a featuring a unique nanoring architecture, is designed for highly efficient and durable OER in alkaline media. Operando spectroscopic analyses combined with density functional theory calculations reveal a pronounced element‐specific synergy within the medium‐entropy framework. High‐valence Co species act as the primary catalytic centres, while Fe and Mo cooperatively modulate and stabilize oxidised Co intermediates through dynamic electronic regulation. Meanwhile, Ni largely retains a metallic state, preserving electrical conductivity and structural integrity during operation. The amorphous structure further enhances orbital hybridization, thereby lowering the energetic penalty for O─O bond cleavage and lattice oxygen participation. As a result, FeCoNiMo‐a exhibits outstanding OER activity, requiring an overpotential of only 190 mV to achieve 10 mA cm^−2^ in 1 m KOH, together with excellent long‐term stability. When employed as the anode in an anion exchange membrane water electrolyser (AEMWE), FeCoNiMo‐a delivers current densities of 1 and 5 A cm^−2^ at 1.6 V and 1.9 V, respectively, and maintains stable operation for over 600 h at 1 A cm^−2^. This study elucidates the cooperative mechanisms in medium‐entropy electrocatalysts and provides a rational design strategy for efficient, non‐precious‐metal OER catalysts toward practical alkaline water electrolysis.

## 2 Results and Discussion

### 2.1 Synthesis and Structural Characterization of FeCoNiMo‐a

**FIGURE 1:**
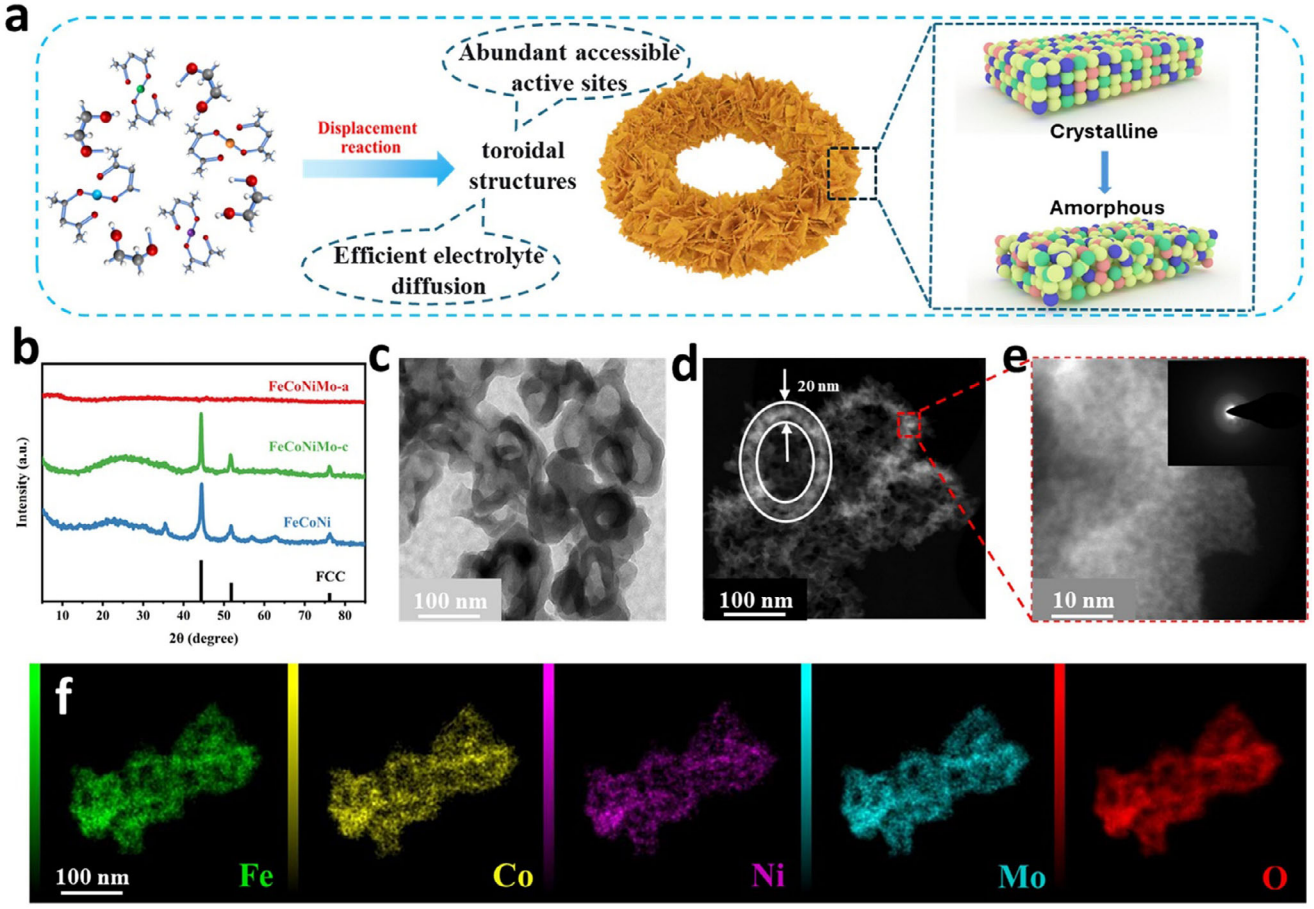
(a) Schematic Diagram of the synthesis of FeCoNiMo‐a. (b) XRD patterns of FeCoNiMo‐a, FeCoNiMo‐c and FeCoNi. (c) TEM image and (d, e) HAADF‐STEM images of FeCoNiMo‐a (The red box is a partial enlargement and the top right corner is the SAED image). (f) EDS mapping of Fe, Co, Ni, Mo, and O elements.

FeCoNiMo MEMs were synthesized via co‐reductant‐assisted solvothermal reduction method (Figure 1a). Specifically, an equal amount of metal acetylacetonate precursors was dispersed in ethanol with ethylene glycol added as a co‐reducing agent. The co‐reducing agents force different metal ions to be reduced simultaneously thereby overcoming their differences in reduction potential to achieve atomic‐level homogeneous mixing. Then the solution was transferred to the autoclave and the product was collected after the reaction. The incorporation of multiple metallic elements with different atomic radii and mixing enthalpies introduces significant chemical and structural disorder, suppressing crystallization and favoring the formation of an amorphous phase with partial configurational entropy contribution. Inductively coupled plasma optical emission spectrometry (ICP‐OES) analysis shows the elemental composition of Fe:Co:Ni:Mo is 0.18:0.22:0.28:0.31 (Table S1). Based on this composition, the calculated mixing entropy using the standard formula (R1) is 1.37R, which satisfies the criterion for classifying this quaternary solid solution as a MEM [34]. We have also prepared a molybdenum‐free reference sample FeCoNi for comparison. The X‐ray diffraction (XRD) spectra in Figure 1b reveal distinct structural differences between FeCoNiMo and FeCoNi. There is no distinct diffraction peak is observed for FeCoNiMo, indicating an amorphous structure. In contrast, the FeCoNi alloy exhibits a series of diffraction peaks at approximately 44.7°, 52.1°, and 76.9°, which correspond to the planes of a face‐centered cubic (FCC) structure. This indicates that the incorporation of Mo suppresses lattice formation in FeCoNiMo MEMs. Moreover, upon high‐temperature treatment, the initially amorphous FeCoNiMo undergoes a pronounced structural transformation. XRD reveals the emergence of characteristic FCC diffraction peaks, indicating thermally induced atomic rearrangement within the amorphous matrix and the consequent formation of a crystalline phase. Accordingly, the samples are denoted as FeCoNiMo‐a and FeCoNiMo‐c, representing the amorphous and crystalline structures, respectively. The elemental compositions of FeCoNi and FeCoNiMo‐c were investigated by ICP‐OES, with the results summarized in Table S1.

Transmission electron microscopy (TEM) image in Figure 1c reveals that the synthesized FeCoNiMo‐a consists of numerous cross‐linked nanorings. These nanorings interconnect to form a three‐dimensional, open network and continuous walls, indicating high structural integrity and controlled nucleation. High‐Angle Annular Dark‐Field Scanning Transmission Electron Microscopy (HAADF‐STEM) further confirms the unique hollow ring‐like nanostructure, showing an average ring diameter of 100 nm and a wall thickness of 20 nm (Figure 1d and Figure S1). Furthermore, the micro‐walls of nanorings are assembled from numerous ultrathin nanosheets, which endows it with a high specific surface area and faster charge transfer channels. Notably, no obvious lattice fringes were observed on the surface of the nanosheets, indicating their amorphous state (Figure 1e). The absence of diffraction rings in the selected area electron diffraction (SAED) pattern further confirms the amorphous nature of the FeCoNiMo‐a (the inset of Figure 1e), which is consistent with XRD results. Amorphous catalysts inherently lack long‐range atomic order, enabling a higher density of unsaturated coordination sites and structural defects [35]. These features effectively expose more accessible active sites, which can significantly enhance OER catalytic activity. The elemental distribution was investigated by aberration‐corrected STEM‐EDS mapping, which clearly demonstrates that all constituent elements (Fe, Co, Ni, Mo, and O) are homogeneously dispersed throughout the nanostructure (Figure 1f and Figure S2). The absence of any detectable phase segregation or elemental enrichment regions indicates successful alloying at the nanoscale and highlights the uniform incorporation of each component during synthesis.

TEM images of FeCoNiMo‐c (Figure S3) show a preserved interconnected nanoring morphology, indicating that high‐temperature treatment does not alter the overall architecture. In contrast, the emergence of well‐defined lattice fringes confirms a structural transition from an amorphous to a crystalline phase induced by thermal treatment. The TEM images of FeCoNi (Figure S4) reveal a markedly different morphology, consisting of a dense, bulk‐like core surrounded by irregular flake‐like edges, rather than the well‐defined nanoring architecture observed in FeCoNiMo‐a. The absence of ring‐like or hollow features suggests that the presence of Mo is essential for directing the formation of the unique nanostructure. This distinction highlights the critical role of Mo, whose larger atomic radius and distinct electronic configuration can modulate nucleation kinetics and atomic diffusion pathways, ultimately promoting hollow structure evolution during synthesis.

The surface areas were evaluated by Brunauer‐Emmett‐Teller (BET) measurements. As shown in Figure S5, the FeCoNiMo‐a catalyst exhibits a high specific surface area of 165.95 m^2^ g^−1^ and a mesopore‐dominated structure with an average pore size of 7.32 nm. FeCoNiMo‐c shows an even higher surface area of 182.4 m^2^ g^−1^ (pore size: 6.30 nm), while FeCoNi has a lower surface area of 121.4 m^2^ g^−1^ and a larger average pore size of 15.08 nm. This finely engineered porous architecture provides abundant active sites and facilitates electrolyte penetration and oxygen release during the OER [36].

X‐ray Photoelectron Spectroscopy (XPS) characterization has been conducted to investigate the surface state of FeCoNiMo‐a. Spectral deconvolution confirms mixed +2/+3 valence configurations for Fe, Co, and Ni, indicative of surface‐oxidized characteristics. This also establishes a multivalent redox reservoir that facilitates electron buffering during catalytic processes. Specifically, the high‐resolution Co 2p spectrum of FeCoNiMo‐a can be deconvoluted into two characteristic doublets, the one located at 780.74 and 796.28 eV corresponds to Co^3+^ species, whereas the other doublet at 782.88 and 797.82 eV is assigned to Co^2+^. An obvious shift to lower binding energy is observed for FeCoNiMo‐c, indicating partial reduction of Co species after high‐temperature thermal treatment [37]. An even more pronounced shift is noted for FeCoNi, which suggests the absence of Mo leads to enhanced electron density at the Co centres. Ni 2p spectra of FeCoNiMo‐a display peaks at 852.67 and 870.11 eV, which are assigned to Ni^0^ [38]. The Fe 2p spectra of FeCoNiMo‐a, FeCoNiMo‐c, and FeCoNi show no significant variation, all exhibiting peaks at 711.25 and 724.12 eV corresponding to Fe^2+^, and at 714.3 and 727.1 eV corresponding to Fe^3+^ [39]. Moreover, the Mo 3d XPS spectrum of FeCoNiMo‐a displays a doublet at 232.47 and 235.6 eV, which is attributed to Mo^6+^, alongside another doublet at 231.66 and 234.9 eV, corresponding to Mo^5+^. In contrast, the spectrum of FeCoNiMo‐c shows two distinct doublets: one at 230.7 and 233.7 eV, assigned to Mo^4+^, and another at 232.47 and 235.6 eV, characteristic of Mo^6+^. This also indicates a partial reduction of the Mo species following high‐temperature thermal treatment [40].

**FIGURE 2:**
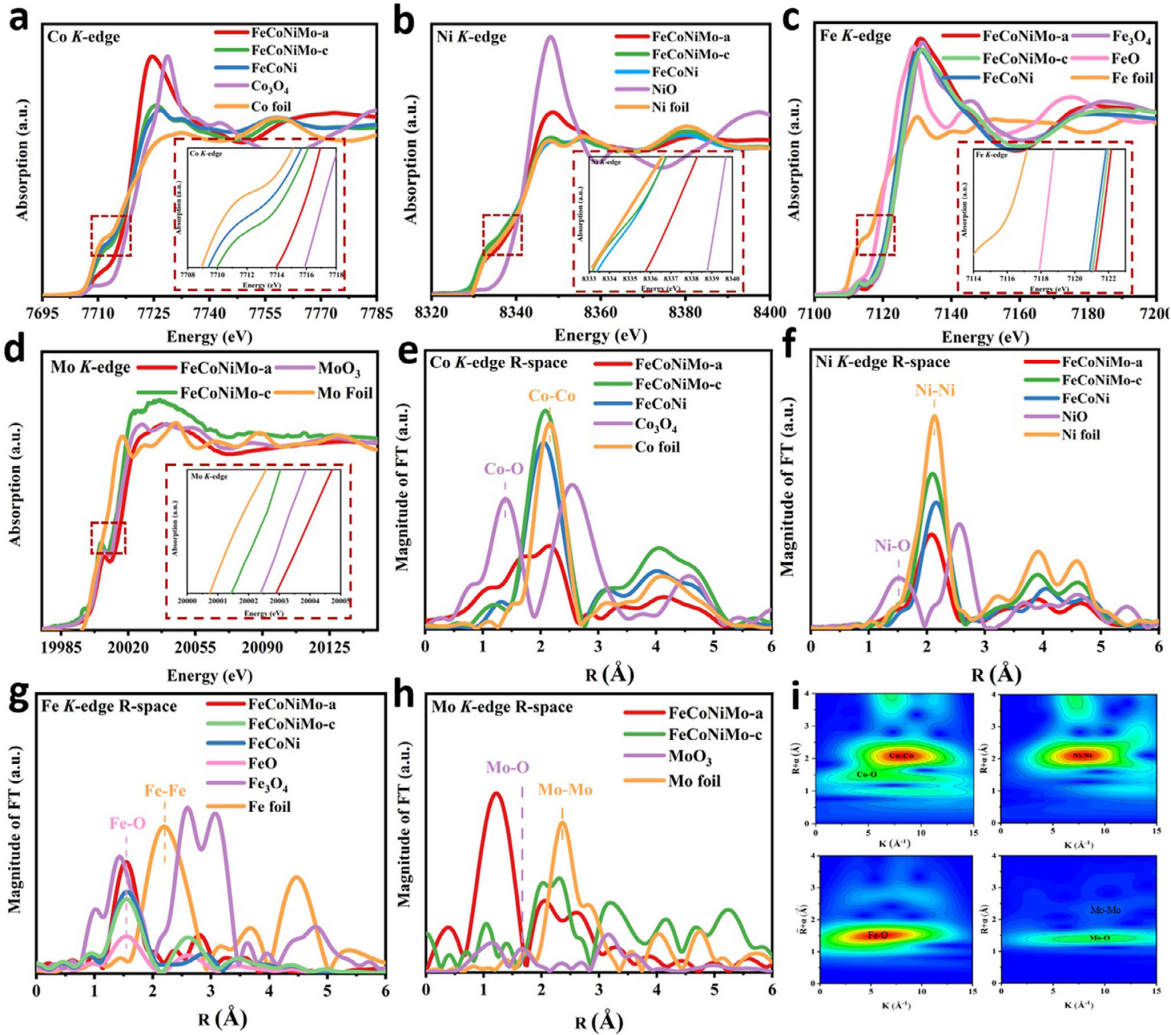
K‐edge X‐ray Absorption Near Edge Structure (XANES) spectra of (a) Co, (b) Ni, (c) Fe, and (d) Mo of different catalysts and samples. R‐space EXAFS spectra at (e) Co K‐edge, (f) Ni K‐edge, (g) Fe K‐edge, and (h) Mo K‐edge of different catalysts and samples. (i) Wavelet transform contour plots of the k^3^‐weighted EXAFS signals for the Co K‐edge, Fe K‐edge, Ni K‐edge, and Mo K‐edge.

We conducted comprehensive X‐ray absorption spectroscopy (XAS) measurements to further investigate the local coordination environments of the metal species. The normalized X‐ray absorption spectra at the K‐edges of Co, Ni, Fe, and Mo are shown in Figure 2a–d, respectively. The insets highlight the near‐edge region around the half‐step height, which is commonly used as a qualitative indicator of oxidation state [41, 42]. As shown in Figure 2a, the absorption edge of Co in FeCoNiMo‐a appears at higher energy than in FeCoNiMo‐c and FeCoNi, albeit still slightly lower than that of Co_3_O_4_, suggesting a relatively higher average oxidation state. A similar trend is observed for Ni in Figure 2b, where FeCoNiMo‐c and FeCoNi exhibit edge positions closer to metallic Ni, whereas FeCoNiMo‐a is shifted to higher energy, indicating a more oxidized state. In Figure 2c, the Fe K‐edge positions for all samples are close to that of Fe_3_O_4_, consistent with an average valence state near +3, with FeCoNiMo‐a showing a slightly higher edge energy. For Mo (Figure 2d), the edge position of FeCoNiMo‐a is comparable to or slightly higher than that of MoO_3_, while FeCoNiMo‐c appears at lower energy, indicating a reduced average Mo valence. Overall, these trends suggest that FeCoNiMo‐a contains more oxidized metal species compared to FeCoNiMo‐c and FeCoNi. This is consistent with the combined influence of Mo incorporation and the amorphous/high‐entropy structure, which may promote partial surface oxidation in agreement with XPS observations.

The local coordination environments were further examined through k^3^‐weighted Fourier transforms (FT) of the EXAFS spectra. In the Co K‐edge R‐space spectra (Figure 2e), FeCoNiMo‐c and FeCoNi both display a dominant Co─Co coordination shell (∼2.1–2.2 Å), characteristic of metallic bonding. In contrast, FeCoNiMo‐a exhibits additional Co─O contributions at lower R (∼1.0–1.5 Å), indicating partial oxidation of Co species induced by Mo incorporation, configurational entropy, and amorphous effects. The Ni K‐edge FT spectra (Figure 2f) show that all samples are dominated by Ni─Ni metallic coordination. Notably, the Ni─Ni peak in FeCoNiMo‐a is slightly shifted to lower R compared to the other samples, which may reflect subtle changes in local bonding (e.g. lattice distortion or alloying effect), rather than a simple bond contraction. As shown in the Fe K‐edge FT (Figure 2g), all samples exhibit a prominent Fe─O coordination shell with no clear Fe─Fe metallic contribution, consistent with Fe predominantly existing in an oxidized environment. A slight elongation in the Fe─O peak position is observed for FeCoNiMo‐a and FeCoNiMo‐c relative to FeCoNi, suggesting modifications to the local structure upon Mo incorporation and amorphization. At the Mo K‐edge (Figure 2h), FeCoNiMo‐c displays a feature at ∼2.3 Å that can be attributed to Mo─Mo coordination. In FeCoNiMo‐a, this feature becomes weaker and shifts to higher R, consistent with a more disordered or oxidized Mo environment, rather than well‐defined Mo─Mo coordination.

Wavelet transform (WT) EXAFS analysis (Figure 2i) further supports these assignments by separating scattering contributions in k‐R space. The results indicate that Fe is primarily coordinated to oxygen, Ni is dominated by metal‐metal coordination, and Co displays a mixed coordination environment involving both Co─O and Co─M contributions. Together, these observations confirm the coexistence of oxidized and metallic bonding environments, with a higher degree of structural disorder in FeCoNiMo‐a.

Based on the above comprehensive structural and compositional characterizations, FeCoNiMo‐a, FeCoNiMo‐c, and FeCoNi are confirmed to possess alloyed metallic cores. Upon exposure to air, their surfaces undergo spontaneous oxidation, leading to the formation of a thin native oxide layer and a characteristic alloy‐oxide core‐shell architecture. This unique structure combines the high electrical conductivity of the metallic alloy core with the catalytic activity of the surface oxide layer, thereby facilitating charge transfer and contributing to the enhanced OER performance.

### 2.2 Electrocatalytic OER Performance

**FIGURE 3:**
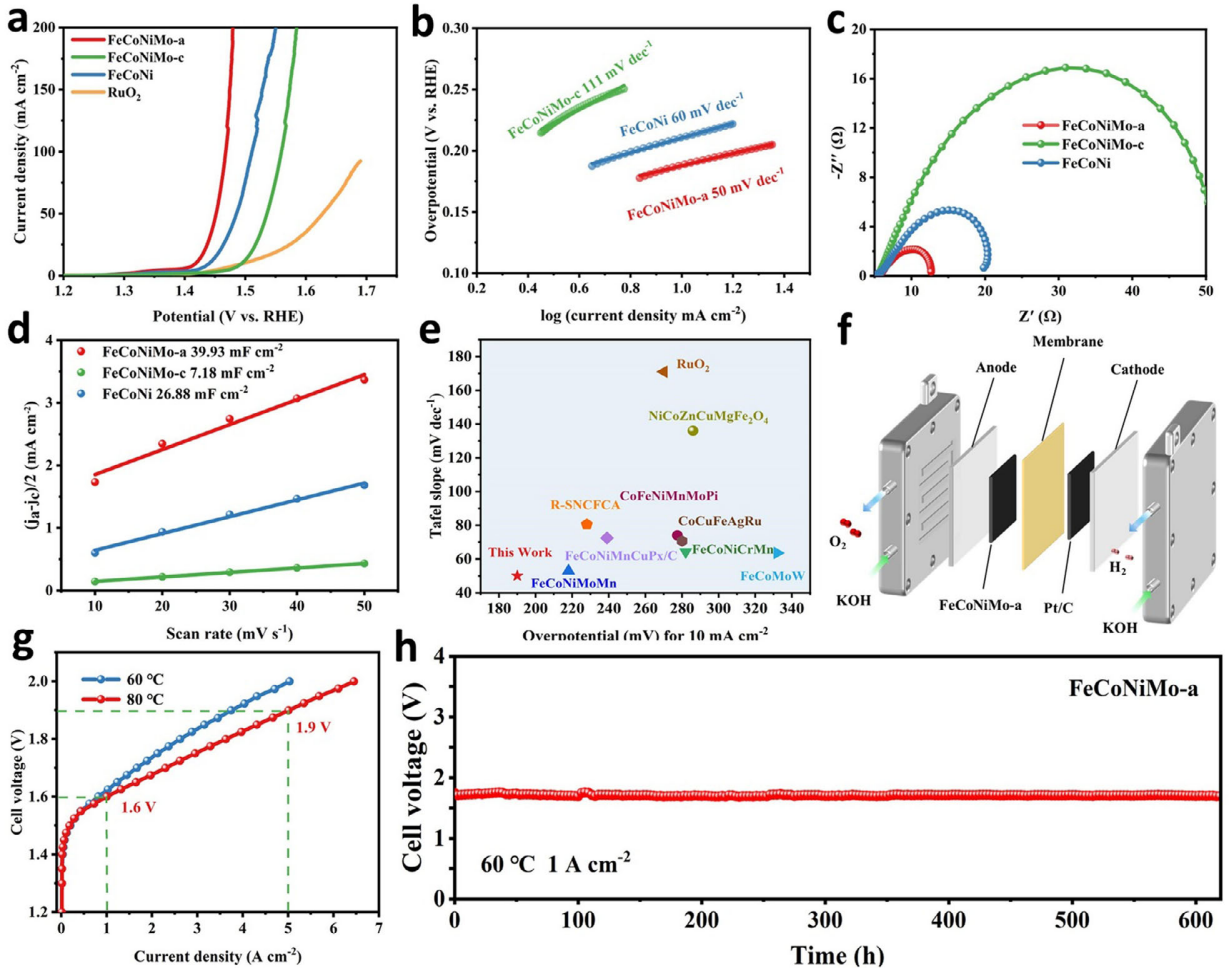
Electrochemical OER performance (a) LSV, (b) Tafel, (c) EIS, and (d) ECSA of FeCoNiMo‐a, FeCoNiMo‐c and FeCoNi. (e) Comparison of overpotentials at a current density of 10 mA cm^−2^ and Tafel slopes for FeCoNiMo‐a and other reported catalysts for OER. (f) Diagram of AEMWE assembly. (g) LSV curves of Pt/C||FeCoNiMo‐a in 1 m KOH at 60°C and 80°C, no iR correction. (h) Stability test result of FeCoNiMo‐a enabled AEMWE at a constant current density of 1 A cm^−2^.

The OER electrochemical performance of FeCoNiMo‐a has been evaluated using a standard three‐electrode system in 1.0 m KOH with carbon paper as the substrate electrode. The bare carbon paper substrate exhibits negligible OER activity (Figure S7). As shown in the LSV results in Figure 3a, FeCoNiMo‐a delivers significantly enhanced OER activity compared to FeCoNi, FeCoNiMo‐c, and commercial RuO_2_. FeCoNiMo‐a shows low overpotentials (η) of 190, 220, and 237 mV to achieve current densities of 10, 50, and 100 mA cm^−2^, respectively, which is much lower than those of FeCoNiMo‐c (260, 300, and 340 mV), FeCoNi (210, 255 and 281 mV), and commercial RuO_2_ (270, 390, and 460 mV). Moreover, FeCoNiMo‐a delivers superior OER activity compared to the crystalline FeCoNiMoO_x_ prepared by annealing FeCoNiMo‐a in air at 400°C (Figure S8), further confirming the superiority of the amorphous alloy structure. Moreover, FeCoNiMo‐a exhibits a low Tafel slope of 50 mV dec^−1^ compared to FeCoNiMo‐c (111 mV dec^−1^) and FeCoNi (60 mV dec^−1^) (Figure 3b). The lower Tafel slope indicates faster reaction kinetics and an effective reaction pathway. The Electrochemical Impedance Spectroscopy (EIS) test results show that FeCoNiMo‐a reveals the lowes charge transfer resistance (R_ct_) value of 6 Ω compared to that of FeCoNiMo‐c (60 Ω) and FeCoNi (15 Ω) (Figure 3c). This indicates that FeCoNiMo‐a allows for much faster charge transfer at the solid‐liquid interface, which is conducive to improving kinetics. The cyclic voltammetry (CV) curves recorded at different scan rates are shown in Figure S9. The electrochemically active surface areas (ECSA) of all samples were determined from the double‐layer capacitance (C_dl_), which was derived from the relationship between (J_a_—J_c_)/2 and scan rate. Among the tested catalysts, FeCoNiMo‐a exhibits the largest ECSA of 39.93 mF cm^−2^, which is 5.56 and 1.48 times to FeCoNiMo‐c and FeCoNi, respectively (Figure 3d). Turnover frequency (TOF) is a key metric to evaluate the intrinsic OER catalytic activity. At 1.45 V vs. RHE, the TOF of FeCoNiMo‐a reaches 0.0595 s^−1^, which is approximately 2.6 times that of FeCoNiMo‐c (0.0227 s^−1^) and 2.2 times that of FeCoNi (0.0276 s^−1^), highlighting its superior intrinsic OER activity (Figure S10). We have also compared FeCoNiMo‐a with other reported MEM catalysts, and it demonstrates outstanding electrochemical activity (Figure 3e and Table S2). In addition to high catalytic activity, FeCoNiMo‐a also demonstrates excellent stability. A negligible decrease in current density was observed after a 50‐h chronoamperometry test, indicating its outstanding long‐term stability (Figure S11).

To assess the practical applicability of the FeCoNiMo‐a electrocatalyst in a real device configuration, an anion exchange membrane water electrolyzer (AEMWE) was assembled using FeCoNiMo‐a/NF as the anode, Pt/C as the cathode, and 1 M KOH as the electrolyte (Figure 3f). The FeCoNiMo‐a/NF‐based AEMWE demonstrates outstanding performance, achieving a current density of 1 A cm^−2^ at a low cell voltage of 1.60 and 1.62 V and requiring only 1.90 and 2.01 V to reach 5 A cm^−2^ at 80 and 60°C, respectively (Figure 3g and Table S3). This further underscores its remarkable intrinsic activity and low kinetic overpotential. Long‐term durability was further evaluated by continuously operating the AEMWE at 60°C and 1 A cm^−2^. Impressively, the electrolyzer exhibits no noticeable voltage decay over 600 h of operation (Figure 3h), demonstrating the exceptional stability and robustness of the FeCoNiMo‐a electrocatalyst under sustained high‐current industrially relevant conditions. These results underscore the strong potential of FeCoNiMo‐a for deployment in practical large‐scale alkaline water electrolysis systems.

### 2.3 OER Catalytic Mechanism

**FIGURE 4:**
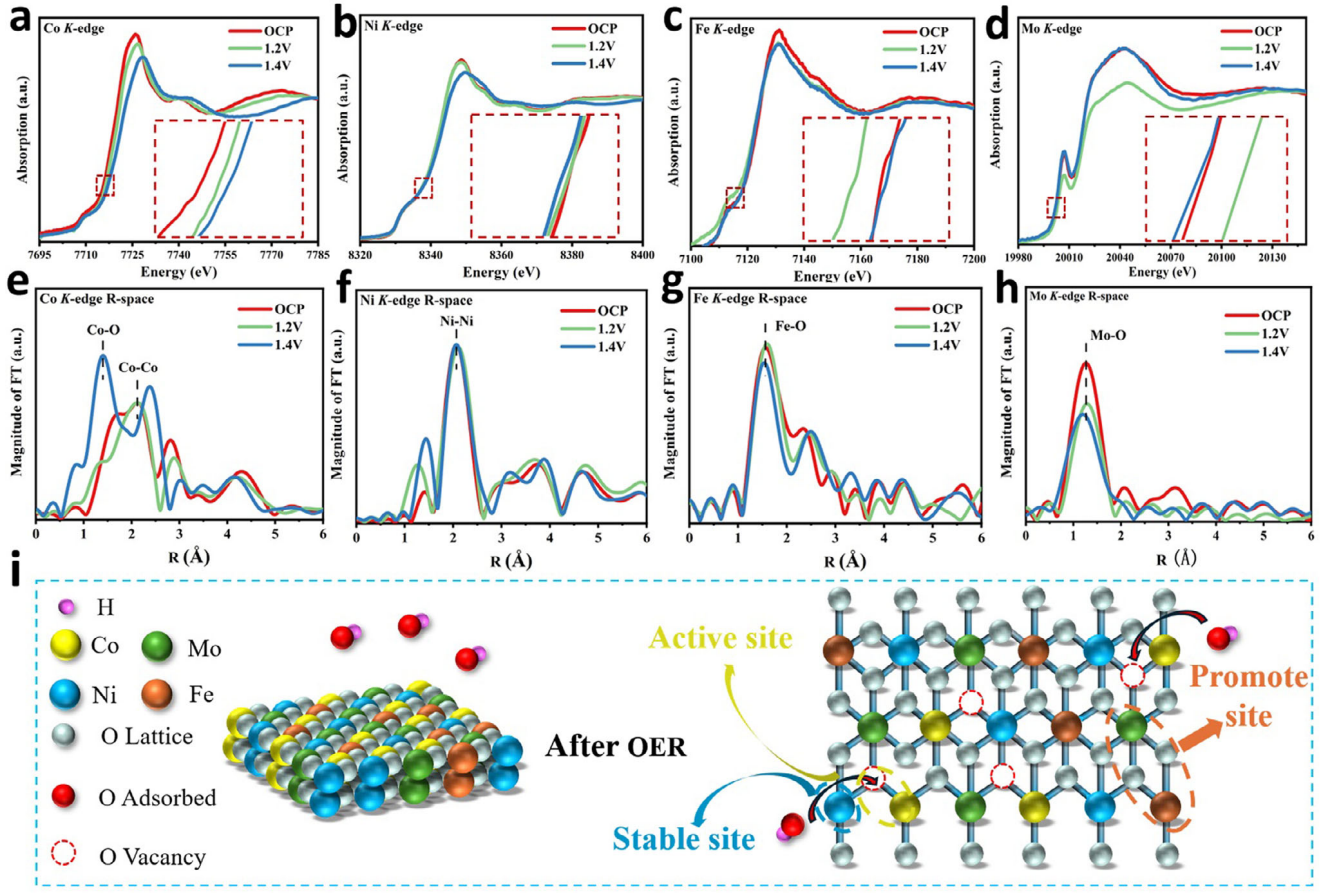
In situ XANES spectra of K‐edge absorption spectra of (a) Co, (b) Ni, (c) Fe, and (d) Mo. R‐space EXAFS spectra at (e) Co K‐edge, (f) Ni K‐edge, (g) Fe K‐edge, and (h) Mo K‐edge of FeCoNiMo‐a. (i) Schematic diagram of phase transition and element role of FeCoNiMo‐a during the OER process.

To investigate the dynamic evolution of the local electronic structure and coordination environment of FeCoNiMo‐a during the OER, we employed in situ XAS to monitor the reaction in real time (Figure 4 and Figure S12). During OER, the Co K‐edge exhibits a progressive shift toward higher energies (Figure 4a), indicating a continuous increase in the oxidation state as Co undergoes electron withdrawal. In contrast, the Ni K‐edge shows negligible change throughout the reaction (Figure 4b), suggesting that Ni maintains a largely stable valence state under operating conditions. As shown in Figure 4c, the Fe K‐edge initially shifts to lower energy before subsequently increasing, resulting in a final oxidation state slightly lower than the initial state. Interestingly, the Mo K‐edge displays an opposite trend to Fe, shifting upward at the beginning of the reaction, and then returning toward lower energy at later stages (Figure 4d).

To further elucidate the evolution of local coordination during OER, Fourier‐transformed EXAFS spectra were analyzed in R‐space to obtain the radial distribution functions of the constituent atoms. As shown in Figure 4e, the Co K‐edge R‐space profiles reveal a progressive change in the coordination environment with increasing potential. Notably, at 1.4 V vs. RHE, the Co coordination structure evolves toward a configuration resembling that of CoOOH, indicating the in situ formation of higher‐valence oxyhydroxide species. For Ni (Figure 4f), the Ni─Ni peak remains dominant at both open‐circuit, 1.2 and 1.4 V vs. RHE, confirming that Ni largely retains a metallic coordination environment. This stable metallic state suggests that Ni primarily serves to maintain high electrical conductivity and facilitate rapid charge transport within the catalyst. In the Fe K‐edge R‐space spectra (Figure 4g), the Fe─O peak shifts to shorter distances at 1.4 V vs. RHE, indicating Fe─O bond contraction and strengthened bond interaction under OER conditions. This enhancement may originate from increased orbital overlap between Fe 3d and O 2p states or from electron depletion at bridging oxygen sites, both of which reinforce Fe─O bonding. Such bond strengthening is expected to contribute to improved structural stability during catalysis. Mo exhibits a similar trend to Fe at 1.4 V vs. RHE, the Mo─O bond undergoes significant contraction, suggesting enhanced Mo─O interactions under oxidative potentials (Figure 4h). This parallel behavior underscores the cooperative role of Fe and Mo in stabilizing the high‐valence oxyhydroxide phases formed during OER.

Therefore, operando XAS analysis during OER provides important insight into their dynamic electronic roles (Figure 4i). The generation of high‐valence Co species constitutes the primary active centers for OER. These oxidized Co states (Co^3+^/Co^4+^) are highly effective in mediating the adsorption and deprotonation of hydroxide ions, promoting O─O bond formation, and ultimately lowering the kinetic barriers associated with oxygen evolution. Ni remains in a relatively low oxidation state and the persistence of dominant metallic Ni─Ni coordination bonds throughout the reaction thereby preserves metallic conductivity, maintains structural integrity, and ensures efficient charge‐transfer pathways. In FeCoNiMo‐a, the incorporation of Fe and Mo constructs an electron‐acceptor environment that effectively stabilizes catalytically active high‐valence Co species. At lower OER potentials, Fe facilitates charge redistribution via Fe─O─Co coupling, promoting the oxidation of Co^2+^ to Co^3+^. At higher potentials, high‐valence Mo functions as a strong electron‐withdrawing center, further shifting and sustaining the cobalt oxidation equilibrium toward higher valence states. This cooperative electronic modulation suppresses the accumulation of Co^2+^, enriches the surface with stable high‐valence Co sites, and enhances redox reversibility. Therefore, Fe and Mo jointly operate as an adaptive electron reservoir, stabilizing Co^3+^ intermediates, and synergistically tuning the local electronic environment of the Co active centers. This cooperative electronic modulation plays a key role in enhancing the intrinsic OER activity of the FeCoNiMo‐a medium‐entropy catalyst.

**FIGURE 5:**
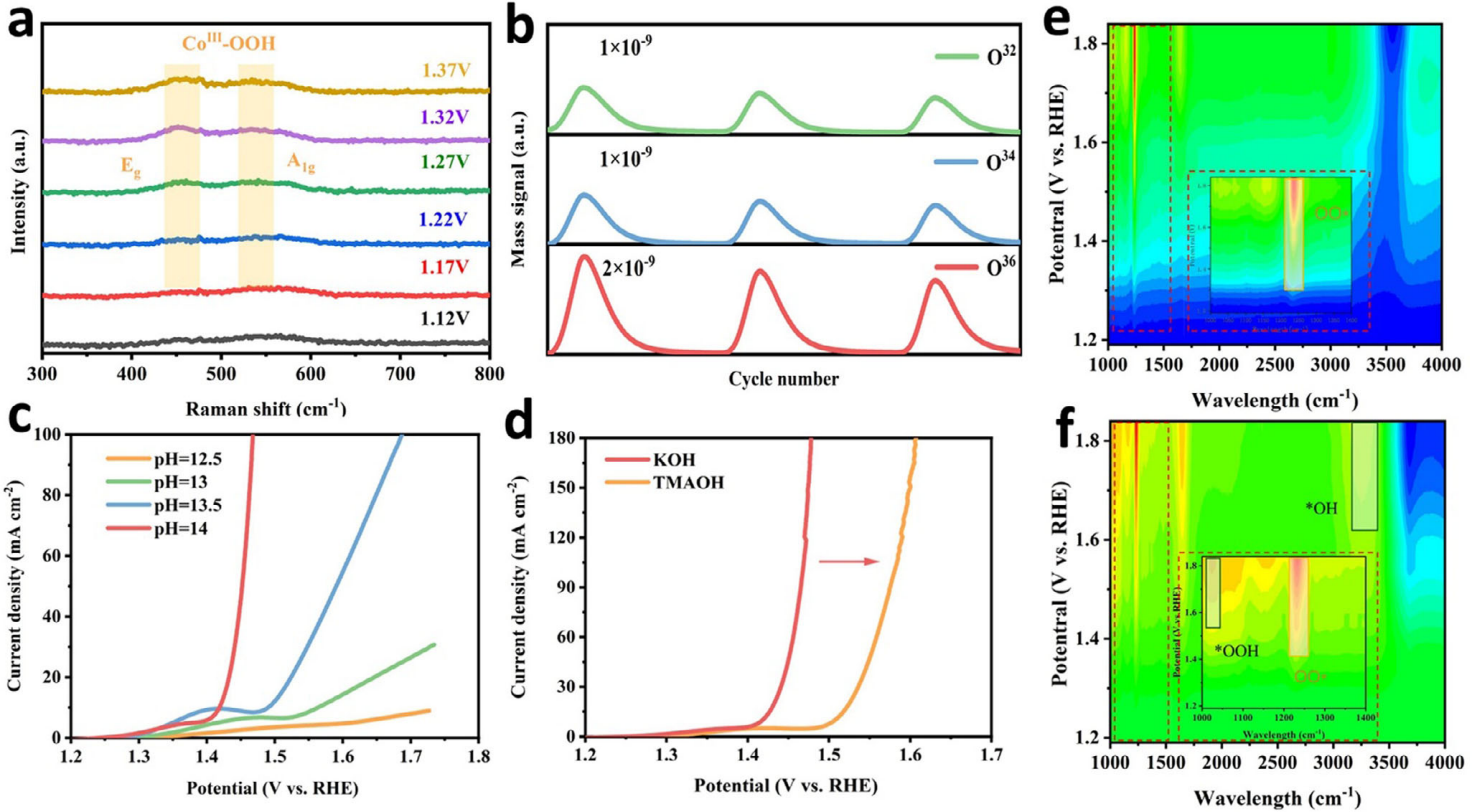
OER Catalytic Mechanism research (a) In situ Raman spectra of FeCoNiMo‐a. (b) DEMS signals of ^32^O_2_, ^34^O_2_, and ^36^O_2_ from the gaseous products for ^18^O‐labeled KOH. (c) LSV curves in KOH solution with pH = 12–14. (d) LSV curves of FeCoNiMo‐a in1.0 m KOH and1.0 m TMAOH. (e, f) In situ ATR‐SEIRAS spectra of FeCoNiMo‐a and FeCoNiMo‐c.

In situ Raman spectroscopy was performed to further elucidate the phase transition behavior of FeCoNiMo‐a under OER conditions. As shown in Figure 5a, characteristic Raman features appear at 452 and 538 cm^−1^, corresponding to vibrational modes of metal‐oxygen bonds in hydroxyl oxide species. The emergence and evolution of these bands confirm a dynamic phase transformation of cobalt from low‐valence states to catalytically active high‐valence oxyhydroxide species (Co^3+^/Co^4+^) during OER, consistent with the formation of the intrinsically active phase observed from in situ XAS results [43].

To further probe the reaction mechanism and associated kinetics, in situ differential electrochemical mass spectrometry (DEMS) was conducted. This operando technique enables sensitive, real‐time detection of gaseous products, thereby providing direct insight into mechanistic pathways and intermediate participation [44]. First, FeCoNiMo‐a was labelled with ^18^O by cycling five times in an ^18^O‐enriched 0.1 m KOH solution. Subsequently, the ^18^O‐labeled material was rinsed with pure water to remove residual H_2_^18^O, followed by cycling in a 0.1 m KOH solution under normal oxygen isotope conditions. The OER was monitored by DEMS, which detected the formation of O_2_ species including ^32^O_2_ (^16^O^16^O), ^34^O_2_ (^16^O^18^O), and ^36^O_2_ (^18^O^18^O). The oxygen generation proceeds via three distinct pathways: (i) from two water molecules (H_2_^16^O) without involvement of lattice oxygen (yielding ^16^O^16^O), (ii) from one water molecule combined with one lattice oxygen atom (^18^O) (yielding ^16^O^18^O), and (iii) exclusively from two lattice oxygen atoms (yielding ^18^O^18^O). Quantitative analysis of the normalized mass spectrometry peak areas (Figure 5b) reveals a pronounced ratio of (^34^O_2_ + ^36^O_2_)/ ^32^O_2_ ≈ 3, indicating that approximately 75% of the evolved oxygen originates from the lattice. This result clearly demonstrates the predominance of the lattice oxygen mechanism (LOM) in FeCoNiMo‐a.

It is well known that LOM involves a non‐coordinated proton‐electron transfer process, and thus LOM‐based catalysts typically exhibit pH‐dependent OER activity [45]. As expected, within the pH range of 12.5–14, the OER activity of FeCoNiMo‐a shows a strong correlation with pH value (Figures 5c and Figure S13). Specifically, its OER activity increases significantly with increasing pH, demonstrating pronounced pH dependence. This suggests that the OER kinetics are likely limited by the non‐concerted proton‐electron transfer (NCPET) process, which is a key signature of the LOM pathway. While FeCoNiMo‐c and FeCoNi also exhibit pH‐dependent OER activity, the dependence is less pronounced compared to that observed for FeCoNiMo‐a. Furthermore, the proton reaction order (ρ^RHE^), which reflects the degree of coupling between proton and electron transfer during OER, was calculated. The results confirm that FeCoNiMo‐a (ρ^RHE^ = 1.12) exhibits the highest degree of decoupled proton‐electron transfer among all samples (compared to ρ^RHE^ values of 0.77 for FeCoNiMo‐c and 0.67 for FeCoNi) (Figure S14) [45]. The high proton reaction order indicates that proton and electron transfer are highly decoupled, suggesting that the reaction proceeds via a NCPET pathway involving lattice oxygen. Since the lattice oxygen‐mediated oxygen evolution process generates negatively charged oxygen‐containing species, the LOM mechanism can be verified through specific electrostatic interactions between TMA^+^ ions and these charged intermediates [46]. When the electrolyte is switched from 1 m KOH to 1 m TMAOH, the overpotential required to achieve 10 mA cm^−2^ increases from 190 to 277 mV, and the Tafel slope rises from 50 to 75 mV dec^−1^, indicating a significant degradation in performance (Figure 5d and Figure S15). This decline is attributed to the strong adsorption of TMA^+^ onto the charged oxygen intermediates, which partially inhibits the OER. These observations further confirm the operation of the LOM pathway in FeCoNiMo‐a for triggering the lattice oxygen‐mediated oxygen evolution process. Similarly, suppressed OER activity was also observed for FeCoNiMo‐c and FeCoNi in TMAOH electrolyte (Figure S16), indicating that they likewise follow the LOM mechanism. This conclusion is consistent with the pH‐dependence results described above, collectively providing robust evidence for the LOM pathway in these catalysts. In situ attenuated total reflection surface‐enhanced infrared absorption spectroscopy (ATR‐SEIRAS) is employed to further investigate the OER catalytic mechanism. As shown in Figure 5e, the in situ ATR‐SEIRAS spectra of FeCoNiMoexhibits an absorption band at 1230 cm^−1^, which intensifies with increasing potential. This feature is attributed to the stretching vibration of *O_2_^2−^, a unique reactive intermediate species in the LOM, thereby providing further evidence that the reaction follows the LOM pathway. FeCoNiMo‐c exhibits absorption bands at 1230, 1040, and 3300 cm^−1^, which are typically attributed to *OOH and *OH intermediates (Figure 5f) [47, 48]. This suggests that FeCoNiMo‐c follows a mixed reaction pathway involving both AEM and LOM. FeCoNi exhibits identical in situ IR absorption bands to those of FeCoNiMo‐c, indicating the presence of similar surface‐adsorbed intermediates during operation. This result indicates this stable surface crystalline structure is not conducive to the occurrence of the LOM mechanism (Figure S17).

### 2.4 DFT Calculation

**FIGURE 6:**
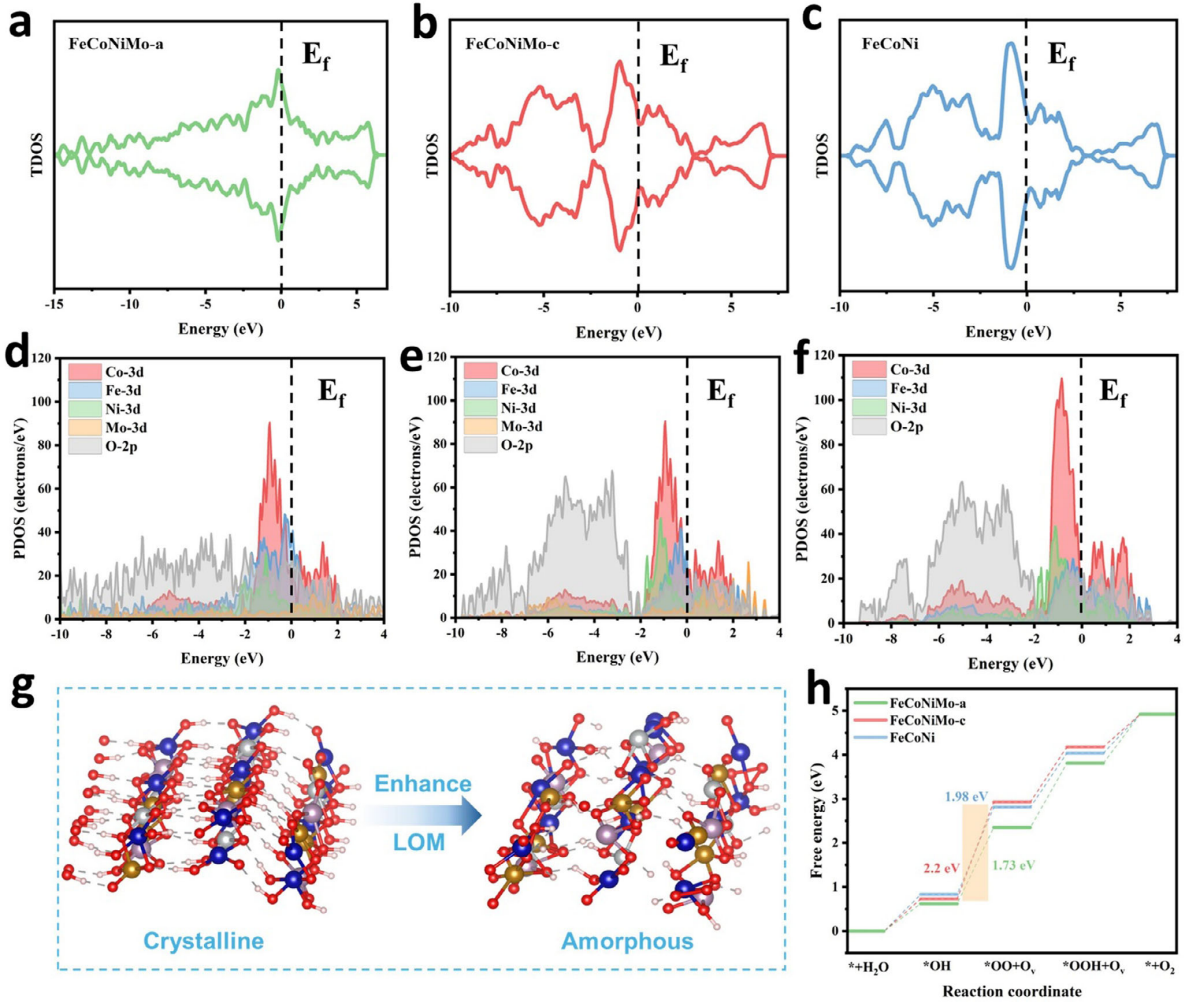
DFT calculations (a–c) Total density of states (TDOS) for FeCoNiMo‐a, FeCoNiMo‐c, and FeCoNi. (d–f) Projected density of states (PDOS) of O 2p and M 3d orbitals for FeCoNiMo‐a, FeCoNiMo‐c, and FeCoNi. (g) Schematic illustration of the promoted LOM process in the amorphous structure, facilitated by active lattice oxygen. (h) Calculated free energy diagrams for the LOM pathway on different catalysts.

To gain deeper insight into the influence of Mo incorporation and the amorphous structure on the OER pathway of FeCoNiMo‐a, density functional theory (DFT) calculations were performed. Based on the structural reconstruction information obtained from in situ XAS and Raman spectroscopy, a cobalt oxyhydroxide (CoOOH) framework was selected as the baseline model for theoretical calculations. In this model, the cobalt sites were randomly substituted by medium‐entropy metal atoms (Fe, Co, Ni, and Mo). Subsequently, an amorphous structure was generated through structural disordering of this model to represent FeCoNiMo‐a. In contrast, the crystalline version of the model was used to represent FeCoNiMo‐c and FeCoNi. Based on the experimental findings presented earlier, the total density of states (TDOS) was calculated (Figure 6a–c). After Mo doping, the DOS near the Fermi level (E_f_) becomes more continuous and enhanced, suggesting improved electronic conductivity. This trend aligns qualitatively with the enhanced charge transfer behavior observed in EIS measurements. Considering that the distance between the O 2p‐band center and E_f_ is a key descriptor for lattice oxygen activity, we computed the projected density of states (PDOS) of O 2p and M 3d orbitals (Figure 6d–f) to elucidate the activity of lattice oxygen in FeCoNiMo‐a. The upward shift of εO‐2p toward Ef in FeCoNiMo‐a indicates enhanced lattice oxygen activity, which may facilitate the LOM [49]. Furthermore, a pronounced overlap between O 2p and M 3d orbitals is observed in FeCoNiMo‐a compared with FeCoNiMo‐c, reflecting stronger orbital hybridization. This increased overlap indicates easier charge transfer, where electrons are no longer localized on the O 2p orbitals but are more evenly distributed across the M─O bonds. Such delocalization reduces the energy barrier for oxygen removal from the lattice and facilitates O─O coupling, which is generally correlated with higher lattice oxygen activity. These results imply that in the amorphous structure, lattice oxygen can participate more readily in the LOM pathway (Figure 6g). To further validate this conclusion, we calculated the energy barriers for key steps along the LOM pathway for FeCoNiMo‐a, FeCoNi, and FeCoNiMo‐c (Figure 6h). LOM pathway typically proceeds through four intermediates (*O, *OOH, *OO, and O_V_), with the initial electrochemical step corresponding to *OH adsorption. Upon Mo incorporation, both FeCoNiMo‐a and FeCoNiMo‐c exhibit optimized *OH adsorption energy compared to FeCoNi, indicating facilitated deprotonation kinetics and more favorable initiation of the LOM process. The subsequent electrochemical step is identified as the rate‐determining step (RDS). FeCoNiMo‐a presents an energy barrier of 1.73 eV, markedly lower than that of FeCoNi (1.98 eV) and FeCoNiMo‐c (2.20 eV). This substantial reduction highlights the decisive role of the amorphous structure in modulating the metal‐oxygen bonding environment and lowering the energetic penalty associated with O─O bond cleavage and lattice oxygen evolution. Consequently, the amorphization synergizes with Mo incorporation to accelerate lattice oxygen activation, thereby promoting the LOM pathway and OER kinetics.

## 3 Conclusion

In summary, we have developed an amorphous quaternary medium‐entropy electrocatalyst, FeCoNiMo‐a, with a nanoring architecture that delivers exceptional activity and durability for oxygen evolution in alkaline media and under practical anion exchange membrane water electrolysis conditions. Beyond performance, this work provides direct insight into how distinct elements cooperate within a disordered medium‐entropy framework. Operando spectroscopic analyses reveal that high‐valence cobalt species serve as the primary catalytic centres, while iron and molybdenum dynamically regulate and stabilize these active intermediates through electronic modulation. In contrast, nickel retains a largely metallic state, preserving electrical conductivity and structural integrity during operation. The amorphous configuration plays a decisive role by enhancing orbital hybridization, thereby lowering the energetic penalty for O─O bond cleavage and lattice oxygen participation. These findings demonstrate that medium‐entropy catalysts can be rationally designed by assigning element‐specific functions rather than relying solely on compositional complexity. More broadly, this study establishes a mechanistic framework for disentangling cooperative effects in multicomponent electrocatalysts and offers design principles for developing efficient, non‐precious‐metal oxygen evolution catalysts toward practical alkaline water electrolysis.

## Conflicts of Interest

The authors declare no conflicts of interest.

## Supporting information




**Supporting File**: advs77381‐sup‐0001‐SuppMat.docx.

## Data Availability

The data that support the findings of this study are available from the corresponding author upon reasonable request.

## References

[1] J. Luo , J.‐H. Im , M. T. Mayer , et al., “Water Photolysis at 12.3% Efficiency via Perovskite Photovoltaics and Earth‐abundant Catalysts,” Science 345 (2014): 1593–1596, 10.1126/science.1258307. 25258076

[2] H. Nishiyama , T. Yamada , M. Nakabayashi , et al., “Photocatalytic Solar Hydrogen Production From Water on a 100‐m^2^ Scale,” Nature 598 (2021): 304–307, 10.1038/s41586-021-03907-3. 34433207

[3] B. Li , J. Zhong , H. Wang , et al., “Fluorine‐Lodged High‐Valent High‐Entropy Layered Double Hydroxide for Efficient, Long‐Lasting Zinc‐Air Batteries,” Angewandte Chemie International Edition 63 (2024): 202410978, 10.1002/anie.202410978. 39287021

[4] F. Fan , B. Lei , X. Song , et al., “Applicable Descriptors Under Weak Metal‐Oxygen d‐p Interaction for the Oxygen Evolution Reaction,” Angewandte Chemie‐International Edition 64 (2024): 202419718, 10.1002/anie.202419718. 39564973

[5] T. Takata , J. Jiang , Y. Sakata , et al., “Photocatalytic Water Splitting With a Quantum Efficiency of Almost Unity,” Nature 581 (2020): 411–414, 10.1038/s41586-020-2278-9. 32461647

[6] Z. Zhu , P. Pei , N. Chen , et al., “Pinning Effect Suppresses Leaching in High‐Entropy Amorphous Alloys for Efficient Selective Electrocatalysis in Alkaline Seawater,” ACS Nano 19 (2025): 38696–38708, 10.1021/acsnano.5c13563. 41160740

[7] H. Xu , Z. Jin , Y. Zhang , et al., “Designing Strategies and Enhancing Mechanism for Multicomponent High‐Entropy Catalysts,” Chemical Science 14 (2023): 771–790, 10.1039/D2SC06403K. 36755717 PMC9890551

[8] J. Kwon , S. Sun , S. Choi , et al., “Tailored Electronic Structure of Ir in High Entropy Alloy for Highly Active and Durable Bifunctional Electrocatalyst for Water Splitting Under an Acidic Environment,” Advanced Materials 35 (2023): 2300091, 10.1002/adma.202300091. 36967600

[9] C. Wang , J. Zhang , K. Miao , et al., “Octahedral Nanocrystals of Ru‐Doped PtFeNiCuW/CNTs High‐Entropy Alloy: High Performance Toward pH‐Universal Hydrogen Evolution Reaction,” Advanced Materials 36 (2024): 2400433, 10.1002/adma.202400433. 38885972

[10] Y. Ma , Y. Ma , Q. Wang , et al., “High‐Entropy Energy Materials: Challenges and New Opportunities,” Energy & Environmental Science 14 (2021): 2883, 10.1039/d1ee00505g.

[11] Y. Yao , Q. Dong , A. Brozena , et al., “High‐Entropy Nanoparticles: Synthesis‐Structure‐Property Relationships and Data‐Driven Discovery,” Science 376 (2022): 6589, 10.1126/science.abn3103. 35389801

[12] L. Sun , K. Wen , G. Li , et al., “High‐Entropy Alloys in Catalysis: Progress, Challenges, and Prospects,” ACS Materials Au 4 (2024): 547–556, 10.1021/acsmaterialsau.4c00080. 39554860 PMC11565283

[13] I. A. Cechanaviciute , R. P. Antony , O. A. Krysiak , et al., “Scalable Synthesis of Multi‐Metal Electrocatalyst Powders and Electrodes and their Application for Oxygen Evolution and Water Splitting,” Angewandte Chemie International Edition 62 (2023): 202218493, 10.1002/anie.202218493. 36640442

[14] Y. Zhang , J. Kang , H. Xie , et al., “Boosting the Oxygen Evolution of High‐Entropy (Oxy)Hydroxide Epitaxially Grown on High Entropy Alloy by Lattice Oxygen Activation,” Applied Catalysis B: Environmental 341 (2024): 123331, 10.1016/j.apcatb.2023.123331.

[15] D. Gao , W. Zhu , J. Chen , et al., “High‐Entropy Effect Promoting Self‐Healing Behavior of Two‐Dimensional Metal Oxide Electrocatalysts for Oxygen Evolution Reaction,” ACS Catalysis 14 (2024): 3700–3711, 10.1021/acscatal.3c05870.

[16] Z.‐J. Chen , T. Zhang , X.‐Y. Gao , et al., “Engineering Microdomains of Oxides in High‐Entropy Alloy Electrodes Toward Efficient Oxygen Evolution,” Advanced Materials 33 (2021): 2101845, 10.1002/adma.202101845. 34250646

[17] F. Wang , P. Zou , Y. Zhang , et al., “Activating Lattice Oxygen in High‐Entropy LDH for Robust and Durable Water Oxidation,” Nature Communications 14 (2023): 1, 10.1038/s41467-023-41706-8. PMC10533845 37758731

[18] M. Long , S. Lai , K. Miao , W. Jiang , W. Fan , and X. Kang , “IrPdCuFeNiCoMo Based Core‐Shell Icosahedron Nanocrystals and Nanocages for Efficient and Robust Acidic Oxygen Evolution,” Angewandte Chemie International Edition 64 (2025): 202419956, 10.1002/anie.202419956. 39632360

[19] P. Rao , Y. Deng , W. Fan , et al., “Movable Type Printing Method to Synthesize High‐Entropy Single‐Atom Catalysts,” Nature Communications 13 (2022): 5071, 10.1038/s41467-022-32850-8. PMC9424199 36038594

[20] Z. Jia , T. Yang , L. Sun , et al., “A Novel Multinary Intermetallic as an Active Electrocatalyst for Hydrogen Evolution,” Advanced Materials 32 (2020): 2000385, 10.1002/adma.202000385. 32267030

[21] B. Zhang , L. Wang , Z. Cao , et al., “High‐Valence Metals Improve Oxygen Evolution Reaction Performance by Modulating 3d Metal Oxidation Cycle Energetics,” Nature Catalysis 3 (2020): 985–992, 10.1038/s41929-020-00525-6.

[22] J. Guo , J. Huo , Y. Liu , et al., “Nitrogen‐Doped Porous Carbon Supported Nonprecious Metal Single‐Atom Electrocatalysts: From Synthesis to Application,” Small Methods 3 (2019): 1900159, 10.1002/smtd.201900159.

[23] S. Gao , S. Hao , Z. Huang , et al., “Synthesis of High‐Entropy Alloy Nanoparticles on Supports by the Fast Moving Bed Pyrolysis,” Nature Communications 11 (2020): 2016, 10.1038/s41467-020-15934-1. PMC7181682 32332743

[24] J. Zhang , Y. Tu , X. Xu , et al., “A High‐entropy Antiperovskite Nitride Enables Efficient Anion Exchange Membrane Water Electrolysis,” Advanced Materials 37 (2025): 2509042, 10.1002/adma.202509042. 40545864

[25] H. Li , Y. Han , H. Zhao , et al., “Fast Site‐to‐Site Electron Transfer of High‐Entropy Alloy Nanocatalyst Driving Redox Electrocatalysis,” Nature Communications 11 (2020): 5437, 10.1038/s41467-020-19277-9. PMC7595151 33116124

[26] Y. Zhao , J. Wu , X. Cao , et al., “High‐Entropy Materials for Water Splitting: An Atomic Nanoengineering Approach to Sustainable Hydrogen Production,” Advanced Materials 37 (2025): 2506117, 10.1002/adma.202506117. 40519044 PMC12422091

[27] H. Zhao , M. Liu , Q. Wang , et al., “Strong Transboundary Electron Transfer of High‐Entropy Quantum‐Dots Driving Rapid Hydrogen Evolution Kinetics,” Energy & Environmental Science 17 (2024): 6594–6605, 10.1039/D4EE01825G.

[28] M. Wei , Y. Sun , J. Zhang , F. Ai , S. Xi , and J. Wang , “High‐Entropy Alloy Nanocrystal Assembled by Nanosheets With d–d Electron Interaction for Hydrogen Evolution Reaction,” Energy & Environmental Science 16 (2023): 4009–4019, 10.1039/D3EE01929B.

[29] S. Liu , Y. Wang , T. Jiang , et al., “Non‐Noble Metal High‐Entropy Alloy‐Based Catalytic Electrode for Long‐Life Hydrogen Gas Batteries,” ACS Nano 18 (2024): 4229–4240, 10.1021/acsnano.3c09482. 38277276

[30] K. Li , J. He , X. Guan , et al., “Phosphorus‐Modified Amorphous High‐Entropy CoFeNiCrMn Compound as High‐Performance Electrocatalyst for Hydrazine‐Assisted Water Electrolysis,” Small 19 (2023): 2302130, 10.1002/smll.202302130. 37345550

[31] A. Sivanantham , H. Lee , S. W. Hwang , et al., “Complementary Functions of Vanadium in Boosting Electrocatalytic Activity of CuCoNiFeMn High‐Entropy Alloy for Water Splitting,” Advanced Functional Materials 33 (2023): 2301153, 10.1002/adfm.202301153.

[32] A. Abdelhafiz , B. Wang , A. R. Harutyunyan , and J. Li , “Carbothermal Shock Synthesis of High Entropy Oxide Catalysts: Dynamic Structural and Chemical Reconstruction Boosting the Catalytic Activity and Stability Toward Oxygen Evolution Reaction,” Advanced Energy Materials 12 (2022): 2200742, 10.1002/aenm.202200742.

[33] C.‐Y. Wu , Y.‐C. Hsiao , Y. Chen , et al., “A Catalyst Family of High‐Entropy Alloy Atomic Layers With Square Atomic Arrangements Comprising Iron‐ and Platinum‐Group Metals,” Science Advances 10 (2024): adl3693, 10.1126/sciadv.adl3693. PMC11277269 39058768

[34] L. Zhang , W. Cai , and N. Bao , “Top‐Level Design Strategy to Construct an Advanced High‐Entropy Co–Cu–Fe–Mo (Oxy)Hydroxide Electrocatalyst for the Oxygen Evolution Reaction,” Advanced Materials 33 (2021): 2100745, 10.1002/adma.202100745. 33876867

[35] G. Wang , Z. Chen , J. Zhu , et al., “High‐Entropy Amorphous Catalysts for Water Electrolysis: A New Frontier,” Nano‐Micro Letters 18 (2025): 77, 10.1007/s40820-025-01936-5. 41111133 PMC12535958

[36] X. Tian , R. Liu , W. Wang , et al., “3D Ordered Macroporous Superstructures of High Entropy Hydroxide With Strong Orbital Coupling Enhancing Water/Seawater Oxidation,” Advanced Materials 37 (2025): 06068, 10.1002/adma.202506068. 40686008

[37] K. Li , J. Zhang , R. Wu , Y. Yu , and B. Zhang , “Anchoring CoO Domains on CoSe_2_ Nanobelts as Bifunctional Electrocatalysts for Overall Water Splitting in Neutral Media,” Advanced Science 3 (2016): 1500426, 10.1002/advs.201500426. 27818899 PMC5071745

[38] T. Wang , G. Nam , Y. Jin , et al., “NiFe (Oxy) Hydroxides Derived From NiFe Disulfides as an Efficient Oxygen Evolution Catalyst for Rechargeable Zn–Air Batteries: The Effect of Surface S Residues,” Advanced Materials 30 (2018): 1800757, 10.1002/adma.201800757. 29782683

[39] Y. Zhao , Z. Shen , J. Huo , et al., “Epoxy‐rich Fe Single Atom Sites Boost Oxygen Reduction Electrocatalysis,” Angewandte Chemie International Edition 62 (2023): 202308349, 10.1002/anie.202308349. 37452696

[40] Y. Huang , M. Li , F. Pan , et al., “Plasma‐Induced Mo‐Doped Co_3_O_4_ With Enriched Oxygen Vacancies for Electrocatalytic Oxygen Evolution in Water Splitting,” Carbon Energy 5 (2023): 279, 10.1002/cey2.279.

[41] Y. Zhao , N. Dongfang , C. Huang , et al., “Operando Monitoring of the Functional Role of Tetrahedral Cobalt Centers for the Oxygen Evolution Reaction,” Nature Communications 16 (2025): 580, 10.1038/s41467-025-55857-3. PMC11723956 39794313

[42] Y. Zhao , D. P. Adiyeri Saseendran , C. Huang , et al., “Oxygen Evolution/Reduction Reaction Catalysts: From In Situ Monitoring and Reaction Mechanisms to Rational Design,” Chemical Reviews 123 (2023): 6257–6358, 10.1021/acs.chemrev.2c00515. 36944098

[43] M. Lee , G. Yoon , M. K. Kim , J. Hong , S. Lee , and G. H. Ryu , “Synthesis of Amorphous Cobalt Hydroxide Nanosheets and Electrochemical Performance According to Phase Transition Into Crystalline Cobalt Oxides,” Journal of Alloys and Compounds 976 (2024): 173282, 10.1016/j.jallcom.2023.173282.

[44] R. Fan , C. Liu , Z. Li , et al., “Ultrastable Electrocatalytic Seawater Splitting at Ampere‐Level Current Density,” Nature Sustainability 7 (2024): 158–167, 10.1038/s41893-023-01263-w.

[45] Z.‐F. Huang , S. Xi , J. Song , et al., “Tuning of Lattice Oxygen Reactivity and Scaling Relation to Construct Better Oxygen Evolution Electrocatalyst,” Nature Communications 12 (2021): 3992, 10.1038/s41467-021-24182-w. PMC8238955 34183651

[46] F. Wang , P. Zou , Y. Zhang , et al., “Activating Lattice Oxygen in High‐Entropy LDH for Robust and Durable Water Oxidation,” Nature Communications 14 (2023): 6019, 10.1038/s41467-023-41706-8. PMC10533845 37758731

[47] Y. Hao , S.‐F. Hung , W.‐J. Zeng , et al., “Switching the Oxygen Evolution Mechanism on Atomically Dispersed Ru for Enhanced Acidic Reaction Kinetics,” Journal of the American Chemical Society 145 (2023): 23659–23669, 10.1021/jacs.3c07777. 37871168

[48] H. Wu , J. Chang , J. Yu , et al., “Atomically Engineered Interfaces Inducing Bridging Oxygen‐Mediated Deprotonation for Enhanced Oxygen Evolution in Acidic Conditions,” Nature Communications 15 (2024): 10315, 10.1038/s41467-024-54798-7. PMC11605066 39609455

[49] F. Wang , L. Feng , M. Zhang , and H. Cong , “Engineering Oxygen Nonbonding States in High Entropy Hydroxides for Scalable Water Oxidation,” Nature Communications 16 (2025): 6624, 10.1038/s41467-025-61766-2. PMC12274509 40681521

