# Supplementary material for: Disentangling Elemental Roles in an Amorphous Medium‐Entropy Electrocatalyst for Oxygen Evolution
Source: Adv Sci (Weinh). 2026 Aug 29:e77381. Online ahead of print. doi: 10.1002/advs.77381 (PMC13525421; doi:10.1002/advs.77381)
Supplement: Supplementary file 1 [file ADVS-9999-e77381-s001.docx]

**Supporting Information**

**Disentangling Elemental Roles in an Amorphous Medium-Entropy Electrocatalyst for Oxygen Evolution**

Jinhu Wu^[a], [b]^, Xianjun Cao^[a]^, Cheng Gong^[a]^, Dongfang Li^[b]^, Yaojie Lei^[b]^, Daojin Zhou^[c]^, Pengpeng Zhang^[a]^, Zeliang Wu^[b]^, Bin Yu^[a], [b]^, Xingwang Peng^[a]^, Bernt Johannessen^[d]^, Somnath Chanda Roy^[e]^, Jinqiang Zhang*^[b]^, Hao Liu*^[b]^, Yufei Zhao*^[b]^

[a]	Jinhu Wu, Xianjun Cao, Cheng Gong, Pengpeng Zhang, Bin Yu, Xingwang Peng
Joint International Laboratory on Environmental and Energy Frontier Materials, School of Environmental and Chemical Engineering.Shanghai University, Shanghai 200444, China.

[b]	Jinhu Wu, Dongfang Li, Yaojie Lei, Zeliang Wu, Bin Yu, Jinqiang Zhang*, Yufei Zhao*, Hao Liu*
School of Mathematical and Physical Sciences, Faculty of Science.University of Technology Sydney. Broadway, Sydney, NSW 2007, Australia.
E-mail: Jinqiang.zhang@uts.edu.au, Hao.liu@uts.edu.au, Yufei.zhao@uts.edu.au

[c]	Daojin Zhou

State Key Laboratory of Chemical Resource Engineering, College of Chemistry, Beijing University of Chemical Technology, Beijing 100029, China.

[d]	Bernt Johannessen

Australian Synchrotron, ANSTO, Melbourne, Victoria, Australia.

[e]	Somnath Chanda Roy

Indian Inst Technol Madras, Dept Phys, Semiconducting Oxide Mat Nanostruct & Tailored Het, Chennai 600036, India.

**Experimental Section/Methods**

**Reagents and materials**

Fe(acac)_3_, Co(acac)_3_, Ni(acac)_2_, MoO_2_(acac)_2_, and ethylene glycol were all purchased from Sinopharm Chemical Reagent Co., Ltd. RuO_2_, ethanol and acetone were purchased from Adamas-beta® Company. Nafion (5%) and Pt/C (20%) were purchased from Sigma-Aldrich Chemical Co., Ltd. All reagents and materials were used directly without any purification process.

**Synthesis of electrocatalysts**

FeCoNiMo-a medium-entropy nanoparticles were synthesized via a hydrothermal method. Metal salts Fe(acac)_3_, Co(acac)_3_, Ni(acac)_2_, and MoO_2_(acac)_2_ were used as precursors, with ethylene glycol serving as the reducing agent. Specifically, 0.2 mmol of each precursor was dispersed in 1 mL of ethanol and stirred for 30 min. The resulting mixture was then transferred to a 25 mL Teflon-lined autoclave, followed by the addition of 15 mL of ethylene glycol. The autoclave was subsequently placed in an oven and maintained at 200 °C for 20 h. After the reaction, the solution was centrifuged, and the precipitate was washed three times with ethanol and acetone, respectively. The obtained material was dried overnight in an oven at 60 °C to yield the powdered product. For comparison, control samples were prepared using similar procedures. FeCoNiMo-c was obtained by annealing the as-prepared FeCoNiMo-a powder in a tube furnace at 400 °C for 4 h under an argon atmosphere. FeCoNi nanoparticles were synthesized following the same hydrothermal method described above, except that no MoO_2_(acac)_2_ precursor was added.

**Material characterizations**

Morphological characterization of the samples was conducted using a Hitachi 7800 transmission electron microscope (TEM) and a JEM-F200 cold field emission transmission electron microscope (CF-TEM). The lattice structure and grain size were further confirmed using the Spherical Aberration-Corrected Transmission Electron Microscope (AC TEM-JEOL GrandARM300F2). The crystallinity and phase composition of the catalysts were investigated using a D/MAX2200-3kW powder X-ray diffractometer. Inductively coupled plasma optical emission spectroscopy (ICP-OES) was performed on an iCAP PRO instrument. X-ray photoelectron spectroscopy (XPS) measurements were carried out using an ESCALAB 250Xi instrument (Al Kα radiation, Thermo Fisher Scientific, USA). Calibration of the XPS spectra was conducted using the binding energy of the C1s peak at 284.8 eV as a reference. In-situ Fourier-transform infrared spectroscopy (FTIR) was performed with a Nicolet iS50 instrument. Raman spectroscopy was conducted using a Lab Ram HR Evolution instrument. Brunauer-Emmett-Teller (BET) surface area and porosity analysis were carried out using an ASAP 2460 instrument. X-ray absorption spectroscopy (XAS) measurements were performed at the Melbourne Synchrotron Radiation Facility at the Co K-edge, Ni K-edge, Fe K-edge, and Mo K-edge.

Calculation formula of configurational entropy based on elemental composition determined by ICP-OES：

$\Delta Smix=-R\sum_{1}^{n} x_{i}lnx_{i}$	Equation R1

**In Situ Characterization Methods**

The detailed in-situ XAS measurements were performed at the Australian Synchrotron (Melbourne). The in-situ XAS spectra were collected using a homemade electrochemical cell (Figure S12) with a three-electrode configuration. The catalyst-loaded carbon paper served as the working electrode, a Ag/AgCl electrode as the reference electrode, and a platinum wire as the counter electrode, with 1.0 M KOH as the electrolyte. Before spectrum collection, a constant potential (OCP, 1.2 V and 1.4 V) was applied for 5-10 minutes to allow the catalyst surface to reach a stable state under the given electrochemical condition. Subsequently, XAS spectrum was collected at each absorption edge (Fe, Co, Ni, and Mo K-edges), with an acquisition time of approximately 15 minutes per element and repeated 3 times.

In situ attenuated total reflectance surface-enhanced infrared absorption spectroscopy (ATR-SEIRAS) measurements were performed to monitor the OER intermediates on the catalyst surface. The experiments were conducted in a custom-built spectroelectrochemical cell equipped with a silicon prism coated with a thin Au film as the substrate. The catalyst was deposited onto the Au-coated Si prism and used as the working electrode. An Ag/AgCl electrode and a platinum wire were used as the reference and counter electrodes, respectively, with 0.1 M KOH as the electrolyte. In situ FTIR spectra were collected at applied potentials of OCP, 1.2 V, 1.4 V, 1.6 V, and 1.8 V vs. RHE. The spectra collected at OCP served as the reference background.

In situ Raman spectroscopy was employed to probe the structural evolution of the catalyst during OER. The measurements were conducted using a confocal Raman microscope with a 532 nm laser excitation source. The catalyst was deposited onto carbon paper and used as the working electrode in a custom-built electrochemical cell. A Ag/AgCl electrode and a platinum wire were used as the reference and counter electrodes, respectively, with 1.0 M KOH as the electrolyte. In situ Raman spectra were collected at applied potentials of 1.12 V, 1.17 V, 1.22 V, 1.27 V, 1.32 V, and 1.37 V vs. RHE.

**Electrochemical measurement**

The electrocatalytic activity of the prepared materials was assessed using a three-electrode system. Hg/HgO electrode, Pt wire electrode, and platinum clip electrode were employed as the reference electrode, counter electrode, and working electrode, respectively. Prior to measurement, the Hg/HgO (1 M KOH) reference electrode was calibrated within the three-electrode system, with two platinum sheet electrodes serving as the working and counter electrodes. The calibration process was conducted in both 1 M KOH and 0.1 M KOH H_2_-saturated electrolytes. An ink loading of 60 μL was applied onto carbon paper and clamped between the platinum clip to function as the working electrode. The ink containing the electrocatalyst was composed of 4 mg of catalyst, 210 μL of ultrapure water, 210 μL of anhydrous ethanol, and 60 μL of Nafion solution (5 wt%). The mixture was sonicated in an ice bath for 3 h. Additionally, a working electrode of RuO2 was prepared. 1 M KOH solution was used as electrolyte. Activation was performed using cyclic voltammetry (CV) with 20 cycles scanned between 1.2 and 1.9 V vs. RHE at a scan rate of 10 mV s-1. Linear sweep voltammetry (LSV) was conducted from 1.2 to 1.9 V vs. RHE at a scan rate of 5 mV s^-1^. All LSV data were iR-corrected using the solution resistance measured by electrochemical impedance spectroscopy (90% compensation, unless otherwise specified).

Using the Nernst equation to convert the E_Hg/HgO_ to E_RHE_:

E_RHE_ = E_Hg/HgO_ + 0.098 + 0.059 * pH	Equation R2

Tafel plots were obtained with low-voltage scans at 1 mV s^-1^. Electrochemical impedance spectroscopy (EIS) was carried out at 1.51 V vs. RHE (1 M KOH). Cyclic voltammetry was performed within the non-Faradaic current range at scan rates of 10 mV s^-1^, 20 mV s^-1^, 30 mV s^-1^, 40 mV s^-1^, and 50 mV s^-1^ to determine the double-layer capacitance of the electrocatalysts.

The turnover frequency (TOF) is a key metric to evaluate the intrinsic catalytic activity of an Oxygen Evolution Reaction (OER) catalyst. We have calculated the TOF using the following equation:

$TOF=\frac{I}{4nF}$	Equation R3

where the factor 4 accounts for the four-electron process involved in O_2_ evolution from water, I is the current (A) at corresponding overpotential from LSV curves, F is Faraday constant (96485 C mol^-1^), n is number of active sites. We assume that all metal sites are active sites for OER, we can derive the following formula:

${TOF}_{surface}=\frac{I N_{A}}{4NmFS_{g}}$	Eqation R4

Where S_g_ is the specific surface area, N represents the number of surface atoms, m is the mass of the load per square centimeter, and NA is Avogadro’s constant.

The long stability test was conducted in a three-electrode system using a constant-current mode. The working electrode, counter electrode, and reference electrode were the same as those in the polarization curve experiment.

In the tetramethylammonium hydroxide (TMAOH) poisoning experiments and pH-dependent tests, LSV performed in TMAOH and KOH solutions with pH values of 12.5, 13, 13.5, and 14, respectively.

The Anion Exchange Membrane Water Electrolyzer (AEMWE) test setup consisted of a conductive layer, a gas diffusion layer, a polytetrafluoroethylene (PTFE) gasket, and an A60 anion exchange membrane. An external pressure of 5 N was applied. The anode was titanium felt loaded with the sample, while the cathode was carbon-based, loaded with Pt/C. Polarization curves were recorded at both 60 °C and 80 °C over a voltage range from 1.2 V to 2.0 V. All measurements were performed without iR compensation. Long-term stability testing was conducted at 60 °C under a constant current density of 1 A cm^-2^ using the chronopotentiometry method for 600 h to evaluate the durability of FeCoNiMo-a.

**Figure S1.** HAADF-STEM images of FeCoNiMo-a.

The extra images further reveal that the structure consists of hollow ring-like nanostructures assembled from ultrathin nanosheets. Additionally, no lattice fringes are observed, indicating an amorphous structure.

**Figure S2.** HAADF-STEM image of FeCoNiMo-a used for EDS mapping.

**Figure S3.** TEM image of FeCoNiMo-c.

**Figure S4.** TEM image of FeCoNi.

**Figure S5.** Adsorption-desorption isotherm of (a) FeCoNiMo-a, (b) FeCoNiMo-c and (c) FeCoNi by BET.

**Figure S6.** The XPS peaks of (a) Co 2p, (b) Ni 2p, (c) Fe 2p, (d) Mo 3d of FeCoNiMo-a, FeCoNiMo-c and FeCoNi.

**Figure S7.** OER polarization curves of the bare carbon paper substrate and the FeCoNiMo-a catalyst loaded on carbon paper in 1.0 M KOH electrolyte.

**Figure S8.** OER polarization curves of the amorphous FeCoNiMo-a and the crystalline FeCoNiMoO_x_ samples in 1.0 M KOH electrolyte.

To further demonstrate the superior OER activity of the amorphous alloy structure, a crystalline FeCoNiMoO_x_ reference sample was prepared by annealing FeCoNiMo-a in air at 400°C. As shown in Figure S8, the FeCoNiMoO_x_ sample exhibits lower OER activity compared to the amorphous FeCoNiMo-a, confirming that the amorphous alloy structure facilitates surface reconstruction and provides more unsaturated active sites for the OER.

**Figure S9.** CV cycles with different sweep speeds of (a) FeCoNiMo-a, (b) FeCoNiMo-c and (c) FeCoNi.

**Figure S10.** TOF of FeCoNiMo-a, FeCoNiMo-c and FeCoNi.

**Figure S11.** Long time stability test of FeCoNiMo-a at 10 mA cm^-2^.

**Figure S12.** Schematic illustration of the in situ XAS experimental setup.

**Figure S13.** pH dependent experiments of (a) FeCoNiMo-c and (b) FeCoNi.

**Figure S14.** Proton reaction order of the OER process of FeCoNiMo-a, FeCoNiMo-c and FeCoNi.

**Figure S1****5.** Tafel slope of FeCoNiMo-a in 1 M KOH and 1 M TMAOH.

**Figure S16.** TMAOH poisoning experiments of (a) FeCoNiMo-c and (b) FeCoNi.

**Figure S17.** *In situ* ATR-SEIRAS spectra of FeCoNi.

**Table S1** The metal contents of FeCoNiMo-a, FeCoNiMo-c and FeCoNi by ICP-OES.

| Sample | Fe (mol%) | Co (mol%) | Ni (mol%) | Mo (mol%) | Fe/M |
| --- | --- | --- | --- | --- | --- |
| FeCoNiMo-a | 20.80 | 25.22 | 32.25 | 21.72 | 0.26 |
| FeCoNiMo-c | 19.93 | 28.34 | 34.45 | 17.28 | 0.24 |
| FeCoNi | 26.5 | 40.54 | 32.96 | / | 0.36 |

**Table S2** Comparison of overpotential at 10 mA cm^-2^ and Tafel slope for FeNiCoMo-a and other catalysts.

| **Electrocatalysts** | **Overpotential (mV) for 10 mA cm^-2^** | **Tafel slope (mV dec^-1^)** | **Ref** | |
| --- | --- | --- | --- | --- |
| FeCoNiMo-a | 190 | 50 | | This work |
| CoFeNiMnMoPi | 277.5 | 74 | | ^[1]^ |
| FeCoNiMoMn | 218 | 53 | | ^[2]^ |
| FeCoNiCrMn | 282 | 64.3 | | ^[3]^ |
| FeCoNiMnCuPx/C | 239 | 72.5 | | ^[4]^ |
| IrPdCuFeNiCoMo NCs/CNTs | 235 | 51 | | ^[5]^ |
| MoZnFeCoNi | 221 | 48 | | ^[6]^ |
| FeCoNiMoMn-LDH | 203 | 53 | | ^[7]^ |
| Li-MnFeCoNiCuHE-LDHs | 195 | 46 | | ^[8]^ |
| (CoCuFeMnNi)_3_O_4_ HEO | 245 | 55 | | ^[9]^ |
| FeZnNNiCoV | 220 | 36 | | ^[10]^ |
| PCFNC@Gr | 280 | 74.9 | | ^[11]^ |
| FeCoNiCrMo-HEAA | 223 | 45.1 | | ^[12]^ |
| RuO_2_ | 270 | 171 | | This work |
| FeCoMoW | 332 | 63.6 | | ^[13]^ |
| CoCuFeAgRu | 280 | 70.7 | | ^[14]^ |
| R-SNCFCA | 228 | 80.52 | | ^[15]^ |
| FeCoNiCuYP/C | 259 | 64 | | ^[16]^ |
| FeCoNiMnCr HEA-HEO | 261 | 42.2 | | ^[17]^ |
| CoFeMnCuZn | 267 | 62.2 | | ^[18]^ |

**Table S3** Comparison of AEMWE Cell voltage at 1 A cm^-2^ for FeNiCoMo-a and other catalysts.

| **Electrocatalysts** | **AEM Cell voltage(V)**  **at 1 A cm ^-2^** | **Ref** |
| --- | --- | --- |
| FeCoNiMo-a | 1.6 | This work |
| IrPdCuFeNiCoMo NCs/CNTs | 1.65 | ^[5]^ |
| MoZn- HEA | 1.91 | ^[6]^ |
| FeCoNiMoMn-LDH | 1.73 | ^[7]^ |
| Li-MnFeCoNiCuHE-LDHs | 1.83 | ^[8]^ |
| (CoCuFeMnNi)_3_O_4_ HEO | 1.75 | ^[9]^ |
| FeZnNNiCoV | 1.8 | ^[10]^ |
| Ti@M-HEO | 1.82 | ^[19]^ |
| PCFNC@Gr | 1.95 | ^[11]^ |
| FeCoNiCrMo-HEAA | 2.18 | ^[12]^ |
| FeCoNiLaPd | 1.67 | ^[20]^ |
| FeCoNiMnCuO HEO | 1.6 | ^[21]^ |

**Supplementary Reference**

[1]	H. Qiao, X. Wang, Q. Dong, et al., “A High-Entropy Phosphate Catalyst for Oxygen Evolution Reaction,” *Nano Energy* **86**, (2021): 106029.

[2]	J. Yuan, J. Chen, Y. Li, et al., “Industrial Robust 2D Multimetallic High-Entropy Oxides for Water Splitting,” *International Journal of Hydrogen Energy* **189**, (2025): 152152.

[3]	P. Yang, Y. Shi, T. Xia, et al., “Novel Self-Supporting Thin Film Electrodes of FeCoNiCrMn High Entropy Alloy for Excellent Oxygen Evolution Reaction,” *Journal of Alloys and Compounds* **938**, (2023): 168582.

[4]	Y. Zhou, C. Feng, M. Chen, et al., “Formation of High Entropy Phosphide Cathode and High Entropy Oxide Anode by Morphology Remodeling for Promoting Hydrogen Production From Overall Seawater Electrolysis,” *International Journal of Hydrogen Energy* **87**, (2024): 771.

[5]	M. Long, S. Lai, K. Miao, et al., “IrPdCuFeNiCoMo Based Core-Shell Icosahedron Nanocrystals and Nanocages for Efficient and Robust Acidic Oxygen Evolution,” *Angewandte Chemie International Edition* **64**, (2025): e202419956.

[6]	Y. Mei, J. Chen, Q. Wang, et al., “MoZn-Based High Entropy Alloy Catalysts Enabled Dual Activation and Stabilization in Alkaline Oxygen Wvolution,” *Science Advances* **10**, (2024): eadq6758.

[7]	D. Liu, P. Guo, X. Yan, et al., “Manipulating the Configuration Entropy of Layered Hydroxides Toward Efficient Oxygen Evolution Reaction for Anion Exchange Membrane Electrolyzer,” *Materials Today* **80**, (2024): 101.

[8]	M. Sun, H. Shang, Y. Shang, et al., “Interstitial Lithium Doping Activates Lattice Oxygen in High-Entropy LDHs for Enhanced and Durable Water Oxidation,” *Advanced Functional Materials* **31**, (2026): e28920.

[9]	H. Kamaruddin, J. Zhang, Y. Lu, et al., “Laser Irradiated Noble Metal-Free High Entropy Oxide 2-D Sheets for Highly Efficient Anion Exchange Membrane Water Electrolyzers,” *Advanced Functional Materials* **35**, (2025): 2507930.

[10]	J. Zhang, Y. Tu, X. Xu, et al., “A High-entropy Antiperovskite Nitride Enables Efficient Anion Exchange Membrane Water Electrolysis,” *Advanced Materials* **37**, (2025): 2509042.

[11]	P. K. L. Tran, T. H. Nguyen, T. T. N. Ta, et al., “Unlocking High-Efficiency Overall Water Electrolysis Through Structurally Ordered High-Entropy Intermetallics-Confined Few-Layered Graphene,” *Advanced Energy Materials* **15**, (2025): e03485.

[12]	Z. Zhu, P. Pei, N. Chen, et al., “Pinning Effect Suppresses Leaching in High-Entropy Amorphous Alloys for Efficient Selective Electrocatalysis in Alkaline Seawater,” *ACS Nano* **19**, (2025): 38696.

[13]	D. Gao, W. Zhu, J. Chen, et al., “High-Entropy Effect Promoting Self-Healing Behavior of Two-Dimensional Metal Oxide Electrocatalysts for Oxygen Evolution Reaction,” *ACS Catalysis* **14**, (2024): 3700.

[14]	Z. Qiu, X. Guo, S. Cao, et al., “High-Entropy Ag−Ru-Based Electrocatalysts with Dual-Active-Center for Highly Stable Ultra-Low-Temperature Zinc-Air Batteries,” *Angewandte Chemie International Edition* **64**, (2025): e202415216.

[15]	Y. Deng, T. Wang, Q. Sun, et al., “Customizing Hetero-Structured High-Entropy Alloy/Oxide Active Site by Regulating the Exsolution Strategy for Highly Efficient Water SSplitting,” *Journal of Alloys and Compounds* **1004**, (2024): 175879.

[16]	X. Li, Z. Xie, S. Roy, et al., “Amorphous High-Entropy Phosphide Nanosheets With Multi-atom Catalytic Sites for Efficient Oxygen Evolution,” *Advanced Materials* **37**, (2025): 2410295.

[17]	J. Hu, T. Guo, X. Zhong, et al., “In Situ Reconstruction of High-Entropy Heterostructure Catalysts for Stable Oxygen Evolution Electrocatalysis under Industrial Conditions,” *Advanced Materials* **36**, (2024): 2310918.

[18]	S. Liu, B. Jia, Y. Wang, et al., “Topological Synthesis of 2D High-Entropy Multimetallic (Oxy)hydroxide for Enhanced Lattice Oxygen Oxidation Mechanism,” *Advanced Materials* **36**, (2024): 2409530.

[19]	C. Lin, Q. Wang, H. Liu, et al., “Direct Laser Writing of Bioinspired High-Entropy Oxide Nanoarrays for Practical Water Electrolysis,” *Advanced Energy Materials* **15**, (2025): e03929.

[20]	Y. Fu, Y. Pan, R. Luo, et al., “Rare-Earth-Metal-Tuned Local Electronic Structure of High-Entropy Alloys for Ampere-Level Electrochemical Overall Water Splitting,” *ACS Catalysis* **15**, (2025): 15509.

[21]	W. Su, T.-J. Huang, H. Huang, et al., “Joule-Heating Synthesis of High-Entropy Oxide Nanoparticles as Sulfion Oxidation Catalysts for Efficient and Durable Hybrid Seawater Electrolysis,” *Advanced Functional Materials* **35**, (2025): 2506415.
